# Analysis of Cameroon’s Sectoral Policies on Physical Activity for Noncommunicable Disease Prevention

**DOI:** 10.3390/ijerph182312713

**Published:** 2021-12-02

**Authors:** Lambed Tatah, Clarisse Mapa-Tassou, Maylene Shung-King, Tolu Oni, James Woodcock, Amy Weimann, Nicole McCreedy, Trish Muzenda, Ishtar Govia, Jean Claude Mbanya, Felix Assah

**Affiliations:** 1 Global Diet and Physical Activity Research Group, Medical Research Council Epidemiology Unit, University of Cambridge, Cambridge CB2 0QQ, UK; tolu.oni@mrc-epid.cam.ac.uk (T.O.); jw745@medschl.cam.ac.uk (J.W.); trishmuzenda@gmail.com (T.M.); 2 Health of Populations in Transition Research Group (HoPiT), University of Yaoundé I, Yaoundé 8046, Cameroon; mapatassou@yahoo.fr (C.M.-T.); jcmbanya@yahoo.co.uk (J.C.M.); kembeassah@yahoo.com (F.A.); 3 School of Public Health and Family Medicine, University of Cape Town, Cape Town 7925, South Africa; Maylene.shungking@uct.ac.za (M.S.-K.); MCCNIC003@myuct.ac.za (N.M.); 4 Research Initiative for Cities Health and Equity (RICHE), Division of Public Health Medicine, School of Public Health and Family Medicine, University of Cape Town, Cape Town 7925, South Africa; amy.weimann@uct.ac.za; 5 African Centre for Cities, University of Cape Town, Cape Town 7701, South Africa; 6 Caribbean Institute for Health Research, The University of the West Indies, Mona Kingston 7, Jamaica; ishtargovia@gmail.com

**Keywords:** physical activity, policy, intersectoral action, noncommunicable diseases, Cameroon

## Abstract

Physical inactivity is increasing in low- and middle-income countries (LMICs), where noncommunicable diseases (NCDs), urbanisation and sedentary living are rapidly growing in tandem. Increasing active living requires the participation of multiple sectors, yet it is unclear whether physical activity (PA)-relevant sectors in LMICs are prioritising PA. We investigated to what extent sectors that influence PA explicitly integrate it in their policies in an LMIC such as Cameroon. We systematically identified policy documents relevant to PA and NCD prevention in Cameroon; and using the Walt and Gilson policy triangle we described, analysed, and interpreted the policy contexts, contents, processes, and actors. We found 17 PA and NCD policy documents spanning from 1974 to 2019 across seven ministries. Thirteen (13/17) policies targeted infrastructure improvement, and four (4/17) targeted communication for behaviour change, all aiming to enhance leisure domain PA. Only the health sector explicitly acknowledged the role of PA in NCD prevention. Notably, no policy from the transport sector mentioned PA. Our findings highlight the need for intersectoral action to integrate PA into policies in all relevant sectors. These actions will need to encompass the breadth of PA domains, including transport, while emphasising the multiple health benefits of PA for the population.

## 1. Introduction

Physical activity (PA) from all domains (occupational, domestic, leisure, and travel) [1] is known to significantly reduce the risk of morbidity and premature mortality and is particularly important for the prevention of non-communicable diseases (NCDs)—mainly cardiovascular diseases, cancers, diabetes, and chronic respiratory diseases—which are rising and cause over 70% of global deaths [2]. Yet levels of insufficient PA remain high globally and are increasing in rapidly urbanising low- and middle-income countries (LMICs). In 2016, 28% of adults worldwide and 18% in sub-Saharan Africa were estimated to have insufficient PA levels [3]. Given that the majority of health behaviours that persist in adulthood are shaped in adolescence [4,5], the fact that over 80% of global adolescents also have insufficient PA levels [3,6] has serious implications for future adult PA behaviour and health [7]. To address this challenge, the World Health Organisation (WHO) has urged countries and communities to take necessary actions to increase their citizens’ PA levels. The WHO’s 2013–2020 Global Action Plan for the Prevention and Control of NCDs highlights PA as a priority area [8]; and the Global Action Plan for Physical Activity (GAPPA) 2018–2030: more active people for a healthier world, outlines in detail the steps needed to increase PA [8].

The prevention of NCDs is particularly crucial for LMICs where three-quarters of all NCD-related deaths, four-fifths of NCD premature deaths, and the highest loss of disability-adjusted life years occur [2]. In addition, LMICs suffer significant human, social and economic consequences from NCDs, with unaffordable costs of disease management for most individuals, families, communities, and states. The decrease in economic productivity associated with NCDs also perpetuates the vicious cycle of poverty [9]. The predominance of communicable diseases, poverty, weak health systems, conflicts, and governance issues in LMICs makes the control of NCDs more challenging and prevention more urgent. 

Cameroon, similar to most LMICs, has a weak, underfunded health system that is overwhelmed by communicable diseases such as HIV/AIDS, malaria and tuberculosis, and high maternal and child mortality [10]. It also faces challenges in governance, conflict and insecurity that are common in many LMICs. These challenges present opportunity costs to building healthy, active, and sustainable communities. A 2014 NCD report showed that NCDs accounted for 31% of all deaths in the country; with a 20% risk of dying from one of the main NCDs between the ages of 30 and 70 years, and a 35% risk for people aged 50 years and above [11]. The rapid rise of NCDs and their consequences in the country are driven in part by an ongoing socio-economic transition, characterised by improved standards of living, rapid (but mostly unplanned) urbanisation with increasing air and noise pollution [12]. The country’s long term strategic development plan “Cameroon Vision 2035” of 2009 elaborates policy actions aimed at socio-economic transformation, including poverty reduction, industrialisation, and urban growth. The vision also describes how the country’s regional and urban policies contributed to the rapid uncontrolled urbanisation and its associated problems which need addressing. Examples of policy actions that fuelled the observed urbanisation included (i) construction of roads linking most medium size towns and opening of landlocked areas, (ii) setting up of basic social amenities (education, water supply, electricity, hospitals and health centres, telecommunications, and trade centres) in potential towns; and (iii) border area development.

These transitions impact significantly on two key determinants of NCDs, PA and di-et, as more people tend to adopt the consumption of unhealthy and obesogenic diets, as well as shift from an active rural to a sedentary urban lifestyle, associated with insufficient PA [12]. In 2018, it was estimated that a third of Cameroonian adults were not achieving the recommended PA levels, and limited access to safe urban spaces for PA is a purported contributor [3]. Whilst PA and diet are distinct determinants, they are integrally interrelated, and both are contributing to the creation of unhealthy, obesogenic environments that differentially affect different socio-economic groups.

Successfully increasing PA requires collective efforts across different sectors and disciplines, with approaches that are socially and culturally relevant. This calls for the need for intersectoral actions that integrate PA in all relevant sectors of the society while respecting the needs and beliefs of communities. Such efforts should be underscored by messaging to stakeholders that acknowledges and emphasises the importance of PA for NCD prevention and overall health to further bolster the argument for integrating PA goals into all relevant policies. Examples of such intersectoral actions include efforts by the urban planning sector to promote the development of public spaces to encourage PA, and the transport sector to promote safe walking, cycling, and use of public transport in cities. Urban planning and transport are particularly crucial as these influence transport related PA, the second highest contributor to daily PA after work related PA in most LMICs [13]. Developing public spaces to encourage PA aligns with the principle of placemaking, which emphasises the collective reimagination and reinvention of public spaces as the heart of every community and city [14]. Other key sectors in the PA policy space include the health sector which emphasises the role of PA in the prevention/management of NCDs, as well as the general health and wellbeing benefits of PA; the physical education and sport sector which primarily focuses on promoting sports and physical education; and the education sector that incorporates physical education and sport in most curricula. These holistic intersectoral approaches have been emphasised in the 2018–2030 framework for the implementation of the global action plan on PA in the WHO African region [15].

In Cameroon, as in many LMICs undergoing rapid urbanisation and epidemiological transition, there is limited knowledge on the extent to which different sectors that exert influence on PA integrate the goals of promoting PA and mitigating NCDs into their policies. To address this evidence gap, this study sought to explore the extent to which PA-relevant sectors incorporated PA and NCDs in their policies in Cameroon. This analysis was conducted as part of a broader set of retrospective policy analyses exploring global, African regional, and selected national policy environments for the promotion of PA and diet interventions for NCDs prevention. The analyses form part of the work conducted by the Global Diet and Activity Research (GDAR) network [16], funded through the National Institute for Health Research Global Health Research initiative. The overall aim of the GDAR network is to contribute toward the cocreation of interventions for the reduction of NCDs in LMICs. Other aspects of the GDAR research involve primary data collection to explore lived experiences, environmental exposures, and behavioural risk factors of NCDs.

## 2. Materials and Methods

### 2.1. Study Design

We used an instrumental case study [17] that entailed systematically identifying policy documents relevant to PA and NCD prevention in Cameroon. We then used the Walt and Gilson policy triangle framework, which is widely used for health policy analysis in LMICs and remains a useful framework for gaining insight into the multi-dimensional aspects of policy [18,19], to guide our policy document analysis. It allowed us to describe, analyse, and interpret the policy context, content, process, and actors of PA policies in Cameroon. We focused in detail on the content of these policies to understand whether, and how, they purposively incorporated health goals, as it relates to NCDs. We report our findings in accordance with suggestions from Kayesa and Shung-King on document analysis in health policy analysis studies in low and middle-income countries [20].

### 2.2. Document Search

For this study, we focused on formal, national level Government approved, written policy documents, whilst we recognising that policies comprise much more than formally written documents, and are sometimes expressed as practices, in the absence of written policies. We defined policy documents as Government policies, laws, strategies and plans of action, policy guidelines, policy roundtable discussion reports, policy declarations, statements of intent and other relevant documents. 

**Figure 1:**
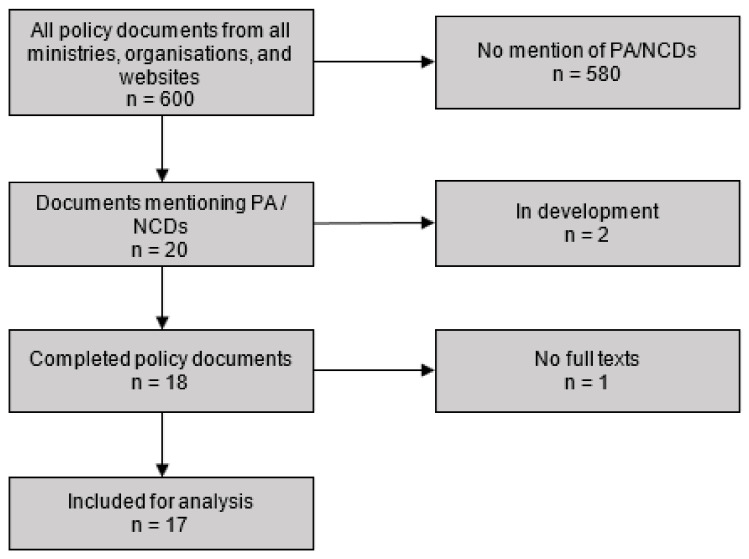
Flowchart of policy documents analysed for PA and NCD policies in Cameroon.

The inclusion and exclusion of policy documents are summarised in Figure 1. Our inclusion criteria for policy documents comprised the following: (i) Written documents that were adopted by June 2020. Because we anticipated few documents, we included all available documents up to the time of the research, June 2020. (ii) Documents that had implications for PA such as addressing issues related to walking, cycling, physical education, and sport. These also included documents that targeted placemaking interventions, i.e., interventions that sought to improve environments through urban design. In addition, included were (iii) documents that were from a PA relevant sector. The PA relevant sectors were identified and purposively selected by the GDAR network senior researchers with experience in PA promotion, based on known (direct and indirect) sector influence on PA and NCD prevention. The following Government sectors, including those specified by the framework for improving PA in Africa [15] were selected: health, housing and urban development, decentralisation (local governance), transport, sport, education, security, culture, entertainment, and youth affairs. In addition, we included cross-sectoral governmental institutions (Offices of the Prime Minister, President, and Parliament), research institutions, non-governmental organisations, and civil society organisations. Our exclusion criteria comprised written documents that were still being developed and not yet adopted, and documents for which the full text could not be found.

In the initial phase of the document search, we considered for screening all the documents that articulated policy intents from all the relevant sectors/ministries. Two researchers, L.T. and C.M.-T. independently hand searched the websites of the relevant ministries between March and June 2020 to compile a list of available policy documents from each sector. During the same search period, relevant databases were queried for articles and reports that referenced relevant policy documents in Cameroon. Databases included PAIS Index, Sabinet Legal database, PubMed, Google Scholar and Science Direct databases and the Google search engines. We used various combinations of the following search terms to identify sentinel documents on the internet search: “non-communicable diseases”, “NCDs”, “cancer”, “diabetes”, “heart disease”, “obesity”, “NCDs policies”, “physical activity”, “physical inactivity”, “sedentary”, “physical education”, exercise”, “sport,” “walking”, “cycling”, “public transport”, “built environment”, “urban planning”, “Cameroon”, and “health policies”. The search terms were translated into French, and, when the websites had information in both French and English, we conducted searches in both languages. 

The initial list served as a guide for two trained research assistants to retrieve and screen policy documents from the archives of the ministries. The research assistants also asked archive attendants and key informants at the ministries about relevant documents that could meet the inclusion criteria. Our initial search identified 600 policy documents for possible inclusion. Upon screening the document titles, executive summaries (abstracts), and table of contents, 580 did not meet the inclusion criteria (these had no specific relevance to PA and NCDs). Two documents were further excluded because they were still being developed. One document was excluded for lack of full text. Seventeen documents were included for the final data extraction. 

### 2.3. Data Extraction and Analysis

We reviewed full texts of the documents that explicitly mentioned PA or NCDs for data extraction. Data extraction and analysis were iterative. L.T. and C.M.-T. independently coded data into the NVivo 12 software [21]. We combined a top-down (deductive) thematic coding guided by Walt and Gilson policy triangle [18] with a bottom-up (inductive) coding that allowed us to identify new themes not initially thought of as part of the deductive coding framework. The research assistants reviewed full texts of identified policy documents in Government ministries and organisations and identified documents that explicitly mentioned PA or NCDs for data extraction. We based our initial coding on a pre-designed codebook developed for the overarching global policy analysis on diet and physical activity by the Global Diet and Activity Research Network [16]. The following variables were initially coded from each policy document: title, year of publication, level at which document was produced (national, subnational, local), producing agency, primary ‘ownership’ of the document, stated purpose of the document, intended target audience(s) of the document, intended timespan, framing and beliefs specific to PA, PA related NCDs, the role of the state, the policy process, the context, the actors involved, and specific proposals on transport and urban planning (given their significant contribution to the travel domain of PA). We compared and amended our individual extractions.

Based on Walt and Gilson’s policy triangle [18], we analysed, and interpreted the different policy contexts, processes, actors, and content as stated in the policy documents. We used global and regional recommendations on NCDs and PA policies [6,15] to highlight gaps in the policy landscape, as well as identified opportunities for improving policies in Cameroon. The contexts included the country’s relevant socioeconomic and political situation, the challenges of the healthcare system, and the epidemiology of NCDs and PA. The policy process component captured reference to how policy proposals were made, while the actors referred to the stakeholders involved in the policy process. The content referred to any proposals to promote physical activity and/or prevent NCDs, and the implementation and financing of these proposals. The policy proposals were grouped into themes using an inductive process. These themes were based on the targets of the proposals and if the proposal had a level of complexity that necessitated intersectoral actions.

To ensure quality in the research, we held weekly meetings during the project design, data coding, analysis, and write-up phases to agree on data collection tools, and share progress and preliminary findings. We used critiques and insights from other GDAR researchers during these meetings to refine our analysis and synthesis of findings 

## 3. Results

We identified 17 policy documents that explicitly mentioned PA and NCDs in Cameroon (Table 1). The documents spanned from 1974 to 2019 across seven ministries: Office of the Prime Minister, Sport and Physical Education, Health, Decentralisation (Local Governance and Development), Urban Development, Youth Affairs, and Education. The documents included eight laws, three executive orders, five strategic/action plans, and one policy document. Most documents were from the Ministries of Sport and Physical Education (n = 4), Health (n = 4), and Decentralisation (n = 4). While we found 58 policy documents from the transport sector, none of these documents mentioned PA nor NCDs so were not included in the analysis. Seven documents (from the Office of the Prime Minister and Ministries of Decentralisation and Sport and Physical Education) focused on PA as the sole purpose of the document, while the rest of the documents considered actions relevant to PA promotion within actions that addressed sector specific issues. Only one document, the National Integrated and Multi-sector Strategic Plan for the Control of Chronic NCD (NIMSPC-CNCD) of 2011–2015 (Cameroon Ministry of Health, 2010), explicitly focused on NCDs. We describe the contexts, actors, processes, and contents of these policies below.

### 3.1. Context

The local social, cultural, economic, health, and political contexts that influenced PA related policies in Cameroon varied the four decades covered by the documents. In general, policies converged toward addressing infrastructural barriers and contextual needs for promoting culture, health and wellbeing, and competitiveness in sports. Expressions of PA were largely confined to physical education, exercise, and sport. A temporal evolution in PA policies was discernible, where a shift from focusing on competitive sport to include exercise and physical education and then health was observed.

*“ARTICLE 1—(1) Physical activities and sports contribute to the balance in health, education, culture, and development of the individual. They are of a general interest in nature… (Law No. 96/09 of August 5, 1996, establishing the Charter of Physical and Sporting Activities—Sport and Physical Education)”*

[23].

*“Article 18—Traditional games and sports are the expression of the richness of the national cultural heritage (Law No 2018/014 on the promotion of physical activity and sport in Cameroon - Sport and Physical Education)”*

[37].

From a socio-economic perspective, underdevelopment, as reflected by the insufficient and uneven distribution of resources and infrastructure, is captured in the background section of a number of policy documents. In the documents the lack of infrastructure is reportedly perceived to be a barrier to the practice of physical activity, especially by youth. The majority of policies therefore sought to address this need through the development of infrastructure for physical education, exercise, and sports. The importance of excelling in sports was expressed as a cultural priority with a desire to display national talents and culture.

*“Youths do not generally practice enough sports and physical education enough because of poor valuation of physical education at school, poor enforcement of existing laws, insufficient and uneven distribution of financial, material, and human resources, alongside infrastructural barriers (National Youth Policy 2015—Youth Affairs)”*

[34].

Although health was considered broadly in some policies, it was not until 2010 that NCDs, and their risk factors, were highlighted in health policy documents as needing PA interventions. The 2011–2016 National integrated multisectoral plan for the control of NCDs included a focus on chronic diseases and expressed concerns about the increasing prevalence of NCDs, sedentary living, insufficient infrastructure for PA, rapid urbanisation, and poor implementation of health promotion activities.

*“Urbanization of our cities has resulted in the importation and adoption of certain risk behaviours to cardiovascular disease, the lack of suitable services (development of recreational areas for sports, development health promotion programmes) in our councils. All this will, in years ahead, be one of the causes of the worsening of the current epidemiological situation (NIMSPC-CNCD Cameroon 2011–2016—Health)”*

[30].

*“Health promotion activities are poorly implemented in the country... However, the cost-effectiveness of health promotion interventions on the behaviour change of individuals justifies the effective implementation of the strategic activities of promotion and prevention of CNCDs (NIMSPC-CNCD Cameroon 2011–2016—Health)”*

[30].

The link between the increasing NCD prevalence and different risk factors was also acknowledged, and the 2016–2027 Health Sector Strategy (HSS) emphasised the increasing prevalence of some NCDs and their related behavioural risk factors in the two largest cities in Cameroon. Obesity and overweight, unhealthy diets and insufficient PA were identified as the principal risk factors that needed tackling. The NCD policy document contains clear recommendations for other sectors, including improving public transport for physical activity.

*“Roles of related ministries and stakeholders: Ministry of transport: Implementing policies that limit traffic circulation of private vehicles in urban centres to promote the use of public transport hence encourage physical activities (NIMSPC-CNCD Cameroon 2011–2016—Health)”*

[30].

The contextual perception and framing of the NCD problem and the role of PA in its prevention were found to influence the attention accorded to these diseases. NCDs were framed as a problem in different ways, including as a share of disease burden, disease epidemiological risk factors, and economic consequences associated with the condition. NCDs were also perceived to receive less attention within the health system compared to infectious diseases, such as malaria and HIV, tuberculosis, and maternal and child health. The underfunding of the health systems was perceived to have had a knock-on effect on the failure to prioritise NCD prevention.

*“In the same year, they [NCDs] were responsible for 882 and 862 deaths per 100,000 inhabitants in men and women, respectively. Among the most frequent NCDs are: cardiovascular diseases, cancers, road accidents (National Health Development Plan 2016–2020.Cameroon—Health)”*

[36].

*“The current health situation is characterized by the predominance of communicable diseases (HIV/AIDS, malaria, tuberculosis, etc.) and a significant increase of non-communicable diseases, including cardiovascular conditions, cancers, mental diseases, and trauma due to road accidents (National Health Development Plan 2016–2020. Cameroon—Health)”*

[36].

### 3.2. Actors

Documents from the Office of the President and Parliament pertaining to the conduct of PA and sports in the country tended to cut across multiple sectors. Similarly, the Office of the Prime Minister, that heads the Government, played a coordinating role in intersectoral actions through executive orders. 

**Figure 2:**
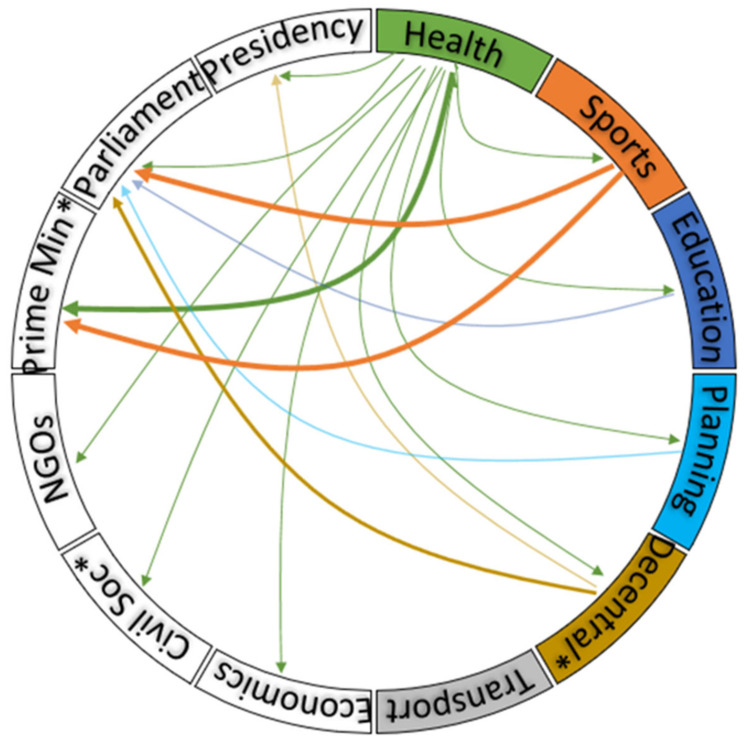
Main actors involved in physical activity related policies in Cameroon. Boxes represent the sectors that owned the policies; the arrows point to the sector that collaborated in a policy; and the thickness of the line represents the number of policy documents in which a sector collaborated.

The main sectors involved in policies that influence PA included the Ministries of Sport and Physical Education, Health, Decentralisation (Local Governance and Development), Education, Urban Planning, and Youth Affairs. Figure 2 shows the main sectors and with whom they collaborated in the development of policies. The transport sector did not have policies that mentioned PA. The health sector involved multiple sectors in the development of its strategic plans, although the participating sectors did not have corresponding plans reflecting their engagement with the Ministry of Health.

### 3.3. Processes

As expected, most policy documents did not explicitly outline the processes involved in policy development. Laws, which make up the majority of the documents, would have been deliberated in parliament before being signed by the President, but information was not available in the documents that could enable tracing the sectors involved in the working group. Similarly, executive orders issued by the Prime Minister’s Office were mainly informed by existing policies. While their development may have been instigated by the ministries concerned, this was not documented. Where documents described the process in detail, policies were developed within frameworks of national and global strategic plans. For example, the National Integrated and Multi-sector Strategic Plan for the Control of Chronic NCD (NIMSPC-CNCD) of 2011–2015 [29] derived directly from the Health Sector Strategy 2001–2015 [25]. Core working groups gathered information and evidence through national surveys, existing studies, and global and regional plans and drafted the initial documents. The initial drafts were finalised in workshops with multi-sectoral teams, including representatives from different ministries and non-governmental organisations in the country, and were adopted by the heads of the different sectors or their representatives.

*“The process of developing the NIMSPC-CNCD [The National Integrated and Multi-sector Strategic Plan for the Control of Chronic NCD] was structured around the following major steps:*

*● Preparation of draft 0 of the NIMSPC-CNCD 2011—2016 by the main actors involved in the fight against NCDs;*

*● Update of the* *NIMSPC-CNCD linked to the 2013—2020 global action plan to combat NCDs;*

*● Conducting a national survey on the prevalence of the main risk factors common to NCDs in Cameroon, STEPS survey, etc.;*

*● Taking into account the results of the STEPS survey in the finalization of the draft of the strategic plan;*

*● The budgetary framework;*

*● The organization of a finalization workshop with a small team;*

*● The organization of a validation workshop by a multisectoral team;*

*● Adoption of the strategy document in the Council of Ministers.*

*(NIMSPC-CNCD Cameroon 2011–2016—Health)”*

[30].

### Global and Regional Policy Reference

Most policy documents drew from global rather than African regional policies. In the elaboration of the 2016–2027 Health Sector Strategy, for example, the objectives were aligned with the sustainable development goals. However, more recent global (NCD and PA) [6] and regional (PA) [15] plans had not yet found expression in any national policy documents.

*“Following the expiration of the MDGs, the UN General Assembly in November 2015 validated new objectives that will guide the development programme of member countries from 2016—2030. The 2016—2027 HSS [Health Sector Strategy] complies with health-related SDGs (SDGs No.3, No.6 and No.13) (Health Sector Strategy, 2016—2027—Health)”*

[35].

New national policies referenced existing national policies for two reasons. Firstly, to describe what had already been done in a sector as seen in the *Growth and Employment Strategy Paper*, where interventions already taken by the State for sports promotion were described.

*“Government… will encourage, within the framework of law n ° 74/22 of 5 December 1970 on sports and socio-educational equipment, of law n ° 96/09 of 5 August 1996 fixing the charter of the physical and sports activities, as well as their texts of application, the creation of sports grounds for mass sport (Growth and Employment Strategy Paper, 2010—all Government policy)”*

[29].

The second reason was to contextualise the policy that was being proposed. For example, in the 2016–2027 Health Sector Strategy, Vision 2035 of the President of the Republic and the *Growth and Employment Paper* were referenced as the overarching direction under which the health sector strategy is being developed.

*“The 2016—2027 HSS [Health Sector Strategy] vision which derived from the 2035 vision of the President of the Republic is formulated as follows: “Cameroon, a country where global access to quality health services is insured for all the social strata by 2035, with the full involvement of communities". To this end, the health sector will work towards contributing to the achievement of the development objectives of the Cameroon Vision by 2035 and the Growth and Employment Strategy Paper (Health Sector Strategy, 2016-2027—health)”*

[35].

### 3.4. Content

Based on our analysis of the policy proposals, two broad categories were addressed across the 17 policy documents: proposals on changes in infrastructure for PA and proposals on communication for behaviour change. The proposals on communication for behaviour change encouraged behaviour change to promote PA through different channels such as advocacy, information, and campaigns without changes in infrastructure or equipment. Proposals on PA infrastructure were those that suggested the provision of sports, exercise, or physical education and other physical infrastructure or equipment to facilitate PA. All the proposals on infrastructure focused on the development of infrastructure that were specific to the promotion of PA without alternative uses. None of the proposals targeted integrated changes in the built environment that align with the principles of placemaking, i.e., collective reimagining and use of public spaces such as roads and public parks. 

Table 2 summarises PA policy proposals identified from different sectors: sport (n = 5), health (n = 4), urban planning (n = 1), local governance (n = 3), education (1), youth affairs (n = 1), cross government (1) and Office of the Prime Minister (1). Thirteen policies (13/17) targeted the improvement of infrastructure that were specific for enhancing physical education, exercise, and sports, and thus leisure domain PA, but no policies were identified that addressed other PA domains such as occupational, domestic, and transport related PA. Four policies (4/17) targeted communication for behaviour change to also enhance leisure domain PA. Policies that explicitly acknowledged the role of PA in the prevention of NCDs were mainly from the health sector.

## 4. Discussion

We explored if and how PA relevant sectors expressed PA in their policies and the level of intersectoral action in the PA and NCD policy spaces in Cameroon. We found that multiple sectors (n = 7) expressed PA in their policies, namely sport, health, local governance, urban planning, education, youth affairs, and the Office of the Prime Minister, although key sectors such as transport and culture made no mention of PA in their policies. A total of 17 policy documents, explicitly expressing PA, were identified; 4/17 proposals targeted the promotion of communication for behaviour change; and 13/17 proposals targeted the development of infrastructure that were specific for the promotion of PA, mainly leisure domain PA. Notably none of the proposals targeting infrastructure considered integrated urban development to promote PA domains such as transport (walking, cycling and use of public transport). The promotion of PA to prevent NCDs and improve health and wellbeing has progressively been acknowledged, although only the health sector has taken an interest in the health benefits of PA. With regard to intersectoral action, many key sectors involved others when undertaking actions relevant to PA, but most of the invited sectors did not mention PA in their own policies. 

The involvement of multiple sectors in the PA policy space in Cameroon speaks to the intrinsic trans-sectoral nature of PA actions, on the one hand, and to the perceived importance of PA, on the other hand. Actions such as the improvement of sport and recreational facilities, road traffic conditions, cultural and social activities, and educational facilities to increase PA are often the direct responsibility of different sectors. PA policies are poorly developed and researched in many LMICs [39]. Filho et al. [40] reported that only few studies in the literature have explored PA interventions in LMICs, mainly noting the importance of school-based, multicomponent interventions. More studies have rather explored multisectoral approaches involved in the promotion of PA related outcomes such as NCDs and general health in LMICs [39,41,42,43]. The multisectoral nature of PA is exemplified in the 2018–2023 Kenyan National Physical Activity Plan, led by the Kenyan Ministry of Health. The plan identifies multiple PA relevant sectors and their responsibilities. In South Africa, just as in Cameroon, multisectoral actions for PA are addressed in the NCD prevention plan and not in a separate national plan for PA. Kang [44] detailed the multisectoral nature of PA in South Korean cities where he examined 393 PA programmes and showed that 85% of them had some form of multisectoral collaboration.

We found that PA expressions were largely confined to sports, organised exercise, and physical education, which are geared towards improving leisure PA, with policy proposals failing to address other domains of PA such as work and transport related PA (walking, cycling and public transport) that might not only contribute significantly to daily PA levels but also create room for stronger intersectoral actions. Walking, for example, is the dominant mode of transport in Cameroon and other LMICs (mode shares of walking for transport can be as high as 70% in some settings [45]), and an important source of PA. Ignoring the presence and quality of walking environments downplays its importance and encourages people who can afford it to switch to other transport modes [46]. Similarly, cycling has both transport and PA benefits and the lack of cycling infrastructure, such as cycling paths, does not encourage the improvement of the cycling culture in the population [47,48]. Generally, public transport in urban areas contributes to PA mainly through associated walking and cycling. Public transport in Cameroon is described as highly unreliable and unsuitable, with formal buses contributing only 1% of all trips, while commercial motorcycles, which are likely to reduce walking, account for over 60% of motorized urban travel [49]. However, information on travel behaviour in Cameroon is scanty, as is the case with many African countries [50].

The fact that health benefits of PA are not explicitly acknowledged in most policies, except those from the health sector, is a problem, given that policy interventions are partly informed and motivated by perceived benefits or averted harms. The failure to see how PA contributes to health in most sectors therefore limits the sectors’ opportunities to improve PA. Health is a basic human right and the PA’s ability to both improve overall health and prevent NCDs make a relatable case for most sectors. Although it is unclear whether the failure to broadly acknowledge the full spectrum of PA health benefits results from a lack of knowledge, or simply reflects the sector’s area of priority, an intersectoral approach to policy making for improved PA could help in both cases, especially the former, where it leads to increased understanding of potential policy benefits. Kahan argues that a condition is recognised as a social issue when people present information about it in a way that leads society to believe that the condition is important and worthy of attention [51]. It follows that framing appropriate actions for PA requires the basic step of acknowledging its benefits to health. The World Health Organisation recognises that the health ministries, rather than other ministers or heads of Government, will often need to lead intersectoral initiatives for health and suggests practical steps such as illustrating the health impact of policies administered by other ministries, and the health benefits of a collaborative approach to policy development [52]. 

With regard to approaches to intersectoral actions for the promotion of PA, our findings showed two key strategies: the cross-government sectors such as the Office of the Prime Minister organising interministerial committees to tackle PA related intervention [28], and ministries identifying and collaborating with relevant sister sectors [35]. The creation of intersectoral committees has been recognised as a successful approach [53]; and collaborations among sister sectors directly align with the principles of intersectoral actions as expressed by the WHO from inception in the 1978 Alma Ata declaration [54] and in subsequent declarations including that of the commission on social determinants of health in 2008 [55]. 

Although there are no rules on how intersectoral actions should proceed in any context, two potentially helpful strategies are partially incorporated in the Cameroonian context. Firstly, the parliament/presidency has promulgated multiple pieces of legislation with overlapping and shared common goals which are administered differently across ministries but lacked the intersectoral structures [56] to follow through on this laudable intention. For example, laws on decentralisation and sport could specify intersectoral committees that need to be involved or identify a coordinating ministry. Secondly, the failure to express PA and NCD actions in policies of relevant sectors indicates a lack of consideration of the ‘health in all policies’ approach promulgated by the SDGs and emphasised in the Adelaide Statement on Health in All Policies [57]. Actions of sectors invited to collaborate on PA interventions seemed to be limited to collaboration, as mention of PA could not be traced in most of the sectors. In addition, more structured tools such as the Urban Health Equity Assessment and Response Tool (Urban HEART) [58], which was jointly developed by the World Health Organisation, academia and researchers, and city and national officials in 2010, have been used in over 100 cities in 53 countries to address health inequities through actions on social, economic and physical environmental determinants of health. Both small cities such as Matsapha, Swaziland, with a population of 35,000 and large ones such as Tehran, the Islamic Republic of Iran, with eight million people have used this tool, which in the context of intersectoral action, has directions on how to identify and gather relevant stakeholders [59].

The findings of this study point to the need for Cameroonian NCD and PA related policies to work towards adopting a stronger approach for the development of whole of society policies, health in all policies, and intersectoral policies. As the whole of society policies provide overarching directions for key societal goals and serve as a reference for many sectors, the inclusion of PA objectives in such high-level documents can facilitate their consideration and elaboration downstream. For example, urban health concepts will need to be embedded within national urban development mandates to shift the political will of urban planning-relevant sectors, such as transport, or human settlements, towards intersectoral action to improve health. Relevant PA sectors should also articulate the link between PA and NCDs and health more explicitly in their PA policies. This does not only align with the health in all policies principle but also highlights PA’s contribution to the reduction of NCDs as the global leading cause of mortality and ultimately to the preservation of fundamental human health rights. Furthermore, encouraging different sectors to sit round the table and develop policies on shared problems, including sharing approaches, experiences, and expectations will enhance the intersectoral development of policies. In the case of PA policy, this would mean bringing together the urban planning, health, and transport sectors, among others to discuss needed changes in the urban transport environment. Finally, actors involved in PA policies should consider the full breadth of the PA domains, which will promote a holistic approach to improving PA.

This study has two main strengths. First, it was conducted in tandem with global and regional analyses of policies on NCDs and risk factors conducted by the GDAR [16], which allowed for cross pollination of ideas. As such, the study benefited from an overarching rigorous methodology, iterative feedback from an international steering committee, and real-time comparison of national, regional, and global outcomes to clarify analysis. Second, the context of Cameroon is similar to that of many LMICs, with Cameroon often referred to as Africa in miniature [60]. Our findings would therefore be relevant to a wider context of LMICs undergoing rapid urbanisation and epidemiological transition. Despite filling an important gap in the literature, our study does have limitations. The main limitations of our study are those that are typically associated with case study designs, which includes limited bases for generalising findings and subjectivity in interpreting findings. The specificities of Cameroon could limit the generalisability of our results; however, the country shares many similar geographical, cultural and pollical features with other LMICs that allows for the extension of findings to those settings. We reduced subjectivity in our work by regularly reviewing the research process in team meetings, receiving feedback from the larger GDAR network and external members. A document-based policy analysis also has to contend with the many information gaps that exist in policy documents, but as this is part of a broader research agenda, the complimentary information on the lived experiences of citizens of PA and other NCD determinants are addressed in other parts of the overarching project.

## 5. Conclusions

We identified multiple sectors involved in the PA policy space in Cameroon, although key sectors such as transport are notably absent. Policies largely focused on physical education, exercise, and sport, which target only leisure PA rather than include work and transport related PA. In addition, the health benefits of PA were not broadly acknowledged, downplaying its importance. Our findings highlight the need for intersectoral action to integrate physical activity into policies in all relevant sectors. These actions will need to encompass the breadth of PA domains including transport and work-related PA while emphasising the health benefits of PA for the population.

## Figures and Tables

**Table 1:** Physical activity related policy documents in Cameroon (until 2020).

Year	Policy Document	Type	Producing Agency	Ownership
1974 ✔	Law No. 74/22 of 5 December 1974, on sport and socio-educational equipment [22]	Law	Parliament	Ministry of Sports and Physical Education
1996 ✔	Law No. 96/09 of 5 August 1996, establishing the Charter of Physical and Sporting Activities [23]	Law	Parliament	Ministry of Sports and Physical Education
1998	Law No. 98/004 of 4 April 1998, on the orientation of education [24]	Law	Parliament	Ministry of Education
2001	Health Sector Strategy 2001–2015 [25]	Strategic document	Ministry of Health	Ministry of Health
2004	Law No. 2004/003 of 21 April 2004 for urban planning in Cameroon [26]	Law	Parliament	Ministry of Housing and Urban Development
2004	Decentralisation law 2004 [27]	Law	Parliament	Ministry of Decentralisation
2008 ✔	ORDER N ° 048 / PM / CAB OF 19 March 2008 on Interministerial Committee for the Supervision of the National Program for the Development of Sports Infrastructures [28]	Executive order	Office of the Prime Minister	Office of the Prime Minister (Including Ministries of Sports, Finance, Planning, Public Works, Land Tenure, Urban Planning and Territorial Administration)
2010	Growth and Employment Strategy Paper (GESP) [29]	Strategic document	Office of the Prime Minister	Office of the Prime Minister (cross- ministry)
2011 *	The National Integrated and Multi-sector Strategic Plan for the Control of Chronic NCD (NIMSPC-CNCD) of 2011–2015 [30]	Action Plan	Ministry of Health	Ministry of Health
2011 ✔	Law No. 2011/018 of 15 July 2011 on the organization and promotion of physical and sporting activities [31]	Law	Parliament	Ministry of Sports and Physical Education
2012 ✔	Decree N° 2012/0881/PM of 27 March 2012 to lay down the conditions for the exercise of certain powers devolved by the State to councils in matters of sports and physical education [32]	Executive order	Office of the Prime Minister	Office of the Prime Minister and Ministry of Sports and Ministry of Decentralisation
2012 ✔	Order No. 14/CAB/PM of 24 September 2012 on organization and functioning of Parcours Vita [33]	Executive order	Office of the Prime Minister	Office of the Prime Minister and Ministry of Sports and Ministry of Decentralisation
2015	National Youth Policy [34]	Policy document	Ministry of Sports	Ministry of Youth Affairs
2016	Health Sector Strategy 2016–2027 [35]	Strategic document	Ministry of Health	Ministry of Health
2016	National Health Development Plan 2016–2020 [36]	Action Plan	Ministry of Health	Ministry of Health
2018 ✔	Law No 2018/014 on the promotion of physical activity and sport in Cameroon [37]	Law	Parliament	Ministry of Sports and Physical Education
2019	Law No 2019/024 of the 24 December 2019 on the general code of decentralized territorial authorities [38]	Law	Parliament	Ministry of Decentralisation

✔ Explicit focus on PA; * explicit focus on NCDs.

**Table 2:** Proposals of physical activity related policies in Cameroon.

Year	Policy Document (Sector)	Communication for Behaviour Change	Physical Activity Infrastructure
1974	Law on Sport and Socio-educational Equipment (Sport) [22]		*“Construction of sports and socio-educational facilities on school, building and societies (industries)”*
1996	Law on Charter of Physical and Sporting Activities (Sport) [23]	Information about “*organization of physical and sport activities, and practice of physical and sport activities (leisure and competition)”*	
1998	Law on the Orientation of Education (Education) [24]		*“The State defines the standards of construction and equipment of public and private educational establishments and ensures their control”*
2001	Health Sector Strategy 2001–2015 (Health) [25]	*“Establishing specific programmes to control obesity and encourage regular physical activity in schools”*	
2004	Law on City Planning in Cameroon (Urban Planning) [26]		*“To construct sports facilities”*
2008	Order on Supervision Development of Sports Infrastructures (Prime Minister) [28]		*“Article 2: The mission of the Committee is to help improve infrastructure and provide sports equipment”.*
2010	Growth and Employment Strategy Paper (GESP) (Cross Government) [29]	Supervision of the sports movement: - *“Provide training in quantity and quality of supervisors* - *Strengthen sports research and excellence centres, promote the organization of competitions in all its dimensions* - *Improve the social protection of athletes and sports professionals* - *Facilitate the functioning of federations, etc”.*	The strengthening of sports governance - *“Sanitation of the sports environment, introduction of good management rules* - *Establishment of a real maintenance policy for existing and future infrastructures* - *Implementation of various incentives for the private sector to invest sustainably and in a multifaceted way in sport* - *The development of sports infrastructures for elite and mass sports.* - *Elite sport: construction of quality sports stadiums will be judiciously constructed and distributed over the national territory, making it possible to deal with the organization of competitions if necessary* - *School sport and the promotion of the practice of sport by the greatest number, the Government will encourage the creation of sports arenas for mass sport”.*
2011	NIMSPC-CNCD 2011–2015 (Health) [30].	*“Key activities: Reinforcement of existing messages and creating new ones for health promotion in order to include major CNCDs risk factors such as unhealthy diet and physical inactivity.”*	
2011	Law on the Organization and Promotion of Physical and Sporting Activities (Sport) [31]		*“Schools, vocational training, higher education and any urban development project must include sports infrastructure and equipment suitable for the practice of physical and sporting activities. The development and management of sports facilities should be organized by the State and local governments”*
2012	Decree on Powers Devolved to Councils in Matters of Sports and Physical Education (Local Governance) [32]		*The State through municipalities is responsible for the creation and the management of sports infrastructures*
2012	Order on Organization and Functioning of Parcours Vita (Sport) [33]		*“Ameliorate the grooming of extra scholar youths by creating socio educative and sporting infrastructure in compliance with urbanization rules; development of proximity infrastructure for the practice of physical education and sports”*
2015	National Youth Policy (Youth Affair) [34]		*“Ameliorate the grooming of extra scholar youths by creating socio educative and sporting infrastructure in compliance with urbanization rules; development of proximity infrastructure for the practice of physical education and sports”*
2016	Health Sector Strategy 2016–2027 (Health) [35]		*“Establishing specific programmes to control obesity and encourage regular physical activity in schools”*
2016	National Health Development Plan 2016–2020 (Health) [36]		*“Strengthening sport and physical activities. Construction/rehabilitation of proximity sport infrastructure for the practice of physical exercise, increase the number of sports instructors in divisions/subdivisions”*
2018	Law on the Promotion of Physical Activity and Sport (Sport) [37]		*“Article 12 (5): the plan for the construction of schools, vocational training and higher education must include sports equipment suitable for the practice of physical and sports activities.”*
2019	Law on the General Code of Decentralized Territorial Authorities (Local Governance) [38]		*“Between the powers transferred to the municipalities, we have the creation and management of municipal stadiums, sports centres and courses, swimming pools, playgrounds and arenas.”*

## Data Availability

Not applicable.
